# SNP Calling, Genotype Calling, and Sample Allele Frequency Estimation from New-Generation Sequencing Data

**DOI:** 10.1371/journal.pone.0037558

**Published:** 2012-07-24

**Authors:** Rasmus Nielsen, Thorfinn Korneliussen, Anders Albrechtsen, Yingrui Li, Jun Wang

**Affiliations:** 1 BGI-Shenzhen, Shenzhen, China; 2 Departments of Integrative Biology and Statistics, University of California, Berkeley, California, United States of America; 3 Department of Biology, University of Copenhagen, Copenhagen, Denmark; University of Montreal, Canada

## Abstract

We present a statistical framework for estimation and application of sample allele frequency spectra from New-Generation Sequencing (NGS) data. In this method, we first estimate the allele frequency spectrum using maximum likelihood. In contrast to previous methods, the likelihood function is calculated using a dynamic programming algorithm and numerically optimized using analytical derivatives. We then use a Bayesian method for estimating the sample allele frequency in a single site, and show how the method can be used for genotype calling and SNP calling. We also show how the method can be extended to various other cases including cases with deviations from Hardy-Weinberg equilibrium. We evaluate the statistical properties of the methods using simulations and by application to a real data set.

## Introduction

The biological sciences have been transformed by the emergence of New-Generation Sequencing (NGS) technologies providing cheap and reliable large scale sequencing (e.g, [1]). These technologies are used for *de novo* genome sequencing (e.g., [2]), in human disease genetics and diagnostics (e.g., [3], [4]), in gene expression analyses (e.g., [5]), in population genetic studies (e.g., [6]), and in many other applications. In this paper, we will mostly be interested in population genetic applications. However, the methods used in this paper may also be helpful for genotype and SNP calling in other studies based on multiple individuals, such as association mapping studies.

Many NGS studies (e.g., [6], [7], [8] are based on medium to low coverage, i.e. coverage at <20X. While the price of NGS is declining, the demand for larger sample sizes is similarly increasing, suggesting that low or medium sequencing coverage may be the design of choice for many future studies in the years to come. In such data, genotype calling for each individual is associated with statistical uncertainty. There are two reasons for this. First, in heterozygous individuals, both alleles may not have been sampled. Secondly, the high raw error rates often associated with NGS may cause a significant amount of homozygous genotypes to be wrongly inferred as heterozygous, if genotype calling is based on just absence/presence of an allele. In most NGS, the error rate is at least 0.1% even after stringent filtering based on quality scores (e.g., [9]). In 5X data, an error will then appear in at least 0.5% of all homozygotes, i.e. at a level comparable to the SNP level. If multiple individuals are sampled, most SNPs will then in fact be errors. For this reason, more stringent criteria are typically used for calling SNPs and for calling heterozygote individuals. Some of these might in effect correspond to requiring the minor allele to be observed twice in an individual to be called. If such a criterion is applied, the chance of calling a heterozygous individual as homozygous in 5X data is approx. 0.375. More clever algorithms can be designed for calling SNPs and for calling genotypes than this (e.g., [10], [11], [12], [13]), but if the coverage is low, they will be sharing the basic features outlined here: a trade-off between including too many SNPs and under-calling true heterozygotes. As a result, low coverage and medium coverage NGS data tends to provide biased estimates of the distribution of allele frequencies ([14], [15], [16], [17] In this paper, we will explore the implications of this for population genetic inferences. We will also present and evaluate a set of algorithms for providing more precise SNP calls, genotype calls, and estimates of allele frequency. The strategy presented in this paper is to estimate the distribution of sample allele frequencies, the so-called Site Frequency Spectrum (SFS), jointly for all individuals and for all sites without calling individual genotypes. When first a good estimate of the SFS has been obtained, better priors can be defined for allele frequencies leading to improved genotype calling and SNP calling. For population genetic inferences, the SFS is in itself of primary interest, and population genetic inferences can proceed directly from the estimated SFS without using individual genotype calls. For example common estimators of effective population sizes and mutation rates, such as Watterson's estimator [18] and π [19] are simple functions of the SFS. Many methods for detecting natural selection, such as Tajima's D [19] are also simple functions of the SFS. Finally, methods for estimating demographic parameters (e.g. [20]) and quantifying population subdivision using *F_ST_* (e.g., [21]) also proceed from estimates of the SFS. For population genetic inferences from next-generation sequencing data, obtaining reliable estimates of the SFS is, therefore, fundamental.

We test the new methods using simulations and apply them to data from 200 previously sequenced human exomes. The methods developed here are available in the program package Analyses of Next-Generation Sequencing Data (ANGSD) downloadable from http://popgen.dk/software/angsd.html.

## Methods

The SFS describes the distribution of allele frequencies. Let the proportion of SNPs, with a derived allele frequency of *i*/*2k* in a sample of *k* diploid individuals, be *p_i_*. The SFS is then given by the vector (*p*_1_, *p*_2_, ... *p*_2*k*-1_). We here consider an expanded version of the SFS: the vector **P** *= * (*p*_0_, *p*_1_, ... *p*_2*k*_), i.e. we also consider sites in the alignment that are fixed. The zero category then represent sites in which all individuals are homozygous for the ancestral allele, and the 2*k* category represents sites that are fixed for the derived allele. The SFS also exists in a so-called folded version, P* = 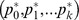, in which 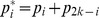 for *i*<*k* and 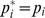 for *i* = *k*. The folded version of the SFS is often used when no reliable information can be used to determine which allele is ancestral and which is derived.

As a note of notation, we distinguish between population allele frequencies and sample allele frequencies by denoting the former by *p*, as in the preceding section, and the latter by *f*. Most of the methods discussed in this paper concerns sample allele frequencies, but we also occasionally discuss the use of population allele frequencies. A number of previous papers have focused on population allele frequencies, including [22], [23]. The methods presented here differ from those methods by focusing on sample allele frequencies, except otherwise stated.

### Calculation of recalibrated quality scores and genotype likelihoods

Any method for SNP calling and allele frequency estimation must rely on a base calling algorithm and a method for calculating quality scores. A quality score is a function of the probability of the most likely base in a particular read given the observed data. It is typically reported using a phred scaling, i.e. as the log_10_ likelihood ratio relative to the most common base. Standard next-generation sequencing methods provide such quality scores associated with each base call. However, the raw quality scores are often not very accurate and must be re-calibrated taking observed error rates in the data into account. The objective of this paper is not to explore different methods for calculating and calibrating quality scores. The methods presented here can be used based on any method for calculating quality scores. However, in our data analyses we use a method similar to the method currently implemented in SOAPsnp [11]. In brief, the raw quality scores from Illumina reads are calibrated taking the observed allelic type and sequencing cycle (coordinate on read) into account. Using observed mismatch rates, the empirical probability of observing the data in a position of a read given the raw quality scores, the sequencing cycle, and the true allelic state can then be calculated. We interpret the probability calculated for read *i* of a particular site as a likelihood of a particular allele, 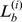, *b* ∈ *B*, *B* = {*A, C, T, G*}. The genotype likelihood, in a site covered by *r* reads, can then be obtained as the product of individual allelic likelihood values (e.g., [24]):


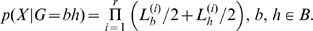
(1)

Notice here that there is an implicit assumption regarding independence of reads in Equation (1). However, this is not the same as assuming Hardy-Weinberg Equilibrium (HWE) as the probability is calculated conditional on the genotype. Posterior probabilities will, in contrast, depend either on HWE assumptions or on an explicit modeling of deviations from HWE. It is also important to notice that the modeling of the error structure in the data is done through the calculation of the genotype likelihood.

### Likelihood function for the allele frequency spectrum

For low coverage data, estimation of allele frequency for a particular site can be associated with great uncertainty. Likewise, SNP calling for rare SNPs can be difficult. However, as shown in the Results section based on methods developed here, the joint estimation for multiple sites in the genome of the distribution of allele frequencies, and the number of SNPs can be carried out with quite high accuracy.

Consider a statistical model in which the sample allele frequencies are free parameters, i.e. for *k* individuals there are 2*k*+1 possible sample allele frequencies including 0 and 1. The vector of parameters is then **P** *= *(*p*_0_, *p*_1_, ... *p*_2*k*_) defined on the unit simplex 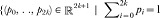 and *p_i_* ≥ 0 for all *i*}. These sample allele frequencies define the SFS with fixed ancestral and derived alleles included. The *i*th sample allele frequency, *p_i_*, is the proportion of sites in the sample in which the derived allele has a frequency of *i*/2k in the sample, *i* = 0,1,..,2*k*. As the sample allele frequencies must sum to one, there are 2*k* parameters to estimate. Estimation of these 2*k* parameters assumes that the ancestral state of each SNP can be identified using outgroups (e.g. other primates for humans). However, if the identification of ancestral state is uncertain, the frequency spectrum can be folded, i.e. the number of observations in category *i* and category 2*k-i* can be added together as described in the results section. For next-generation sequencing data **P** is not known, but must be estimated from the data. An estimate of **P** also provides an estimate of the fraction of variable sites (SNPs) in the sample as 1 – *p*_0_ – *p*_2*k*_. Notice that there is here an implicit assumption that at most two nucleotides are present in the locus. We will later describe how to take into account the presence of more than two nucleotides, but will for now assume that there are at most two alleles, an ancestral allele (a) and a derived allele (A), and that they can be unambiguously identified in each site, except for sites with only one allele.

Assuming that genotype likelihoods can be calculated as discussed above, a likelihood function for **P** can be defined as a function of the genotype likelihood values. Let
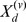
and 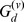 ∈ {0, 1, 2} be the observed data and the unknown genotype, respectively, for individual *d* in site *v*. The genotype counts the number of derived alleles, i.e. 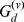 = 0 implies an aa genotype. The genotype likelihood for individual *d* in site *v* can then, with this expanded notation, be written as 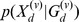. If the genotypes were known, the sampling probability, as a function of **P**, in site *v* would be found by taking the product of the probability of the data given the sample allele frequency multiplied by the probability of the sample allele frequency, given **P**, and then summing over all possible values of the sample allele frequency:


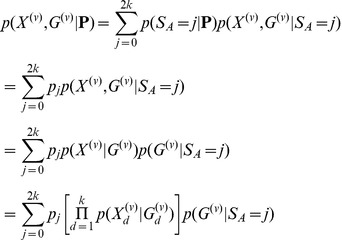
(2)

where the function


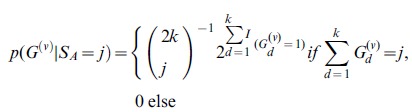
(3)

is the combinatorial probability that a sample contains the labeled genotype vector 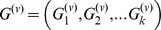 given that it contains a total of *S_A_* alleles of the derived type. This expression assumes Hardy-Weinberg equilibrium.

However, the true genotypes are not known. The likelihood function for **P** must, therefore, be obtained by summing over all the unknown genotypes:


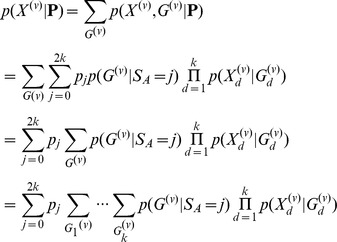


Assuming independence among sites, we then multiply the likelihood among all

sites and obtain:


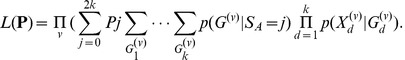
(4)

This likelihood function is the one underlying the EM algorithm applied in [24] and is, if ignoring the difference in handling of errors, also effectively identical to the likelihood function used in [25]. While it might initially appear very challenging to calculate this function for large values of *k* and *v* directly, a simple dynamic programming algorithm can be devised that greatly simplifies these calculations.

### Direct evaluation of the likelihood function

The first step in the algorithm is to calculate the likelihood function for each site, 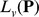, separately. In the following we will describe this algorithm, suppressing the index for site *v* in the notation to enhance readability:

*Initialization:*

Set 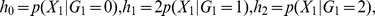
and *h_j_* = 0 for *j* = 3,4,…,2*k*.

*Recursion*

For *d* = 2, 3,…, *k:*

For *j* = 2*d*, 2*d*-1,…,2:

Set
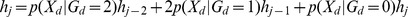


Set *h*_1_ = *p*(*X_d_* | *G_d_* = 0)*h*_1_ + *p*(*X_d_* | *G_d_* = 1)*h*_0_

Set *h*_0_ = *p*(*X_d_* | *G_d_* = 0)*h*_0_

*Termination*

Set 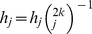
for *j* = 0,1,2,…,2*k.*

The likelihood function can then be expressed as


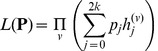
(5)

where 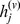
is the value of *h_j_* calculated for the *v*th site (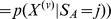. By tabulating the values of 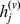
in a table of size (2*k*+1)×*S*, where *S* is the total number of sites, the likelihood function can be re-calculated very fast for different values of **P**. Notice that the computational speed is *O*(*k* ^2^ *S*). Similar algorithms have been used for single site inferences in [8] and [26].

We have here assumed an unfolded (polarized) frequency spectrum. However, the algorithm can also be applied directly to folded data, but with a *k*+1 dimensional parameter space instead of a 2*k*+1 dimensional parameter space.

### Optimization

After tabulation of values of 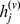
we optimize the likelihood function for **P** using the BFGS algorithm [27]. In order to do that we transform the parameter space from 2*k*+1 to 2*k* parameters. The transformation used is


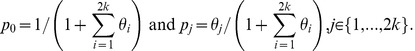
(6)

We then optimize the log likelihood function with respect to the transformed parameters *θ* = (*θ*_1_… *θ*_2*k*_) using analytical derivatives. Application of standard calculus techniques lead to the following derivatives of the log likelihood function for the transformed parameters:


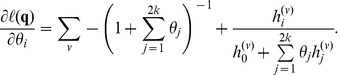
(7)

The BFGS algorithm can then be applied to ***θ***, and the estimates of the natural parameters, **P**, can be found by using the transformation in eq. (6).

### Unknown derived allele

The representation given above assumes that the ancestral and derived (if it exists) alleles always can be unambiguously identified. However, for most next-generation sequencing data, there might be considerable difficulties in separating errors from true low frequency alleles. If the ancestral allele is the common allele, identification of the derived allele will then be ambiguous. The frequency spectrum is only properly defined for di-allelic loci. The approach we will take to this problem is to assume that all loci are truly di-allelic, and errors are responsible when more than two alleles are observed. For most human data, mutation rates are so low that this should be a reasonable approximation. The likelihood function can then be modified by calculating the likelihood for each locus assuming any of the three possible derived alleles, and then adding these likelihood values together, weighted by the probability that each possible derived allele is truly the derived allele. This probability has been set to 1/3 in all analyses presented in this paper. But we note that the inference method could potentially be improved by instead using empirical substitution matrices for this weighting.

We also note that a situation might arise where the inferred ancestral allele is not observed in the data, but two other alleles are segregating, both at high frequency. In these cases the unfolded frequency spectrum is not well-defined. Such loci are typically ignored in population genetic analyses, and will also be ignored here.

### SNP calling and Empirical Bayes estimation of allele frequencies at individual sites

To estimate the sample allele frequency in a single site, we could in theory sum the posterior expectation of the marginal allele frequency calculated for each individual together for all individuals. However, in most applications it will be desirable to obtain the joint posterior distribution for the allele frequency, as downstream inferences then can be performed by integrating over this distribution.

The ML estimates can be used directly in inferences in individual sites for SNP calling, genotype calling, and estimation of allele frequencies. In particular, an Empirical Bayes (EB) method in which the ML estimates are used to make inferences for each individual site might have desirable properties. The posterior probability of the allele frequency in a particular site is given by


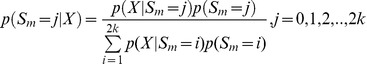
(8)

as in [24] which using the algorithm from the previous section can be calculated as


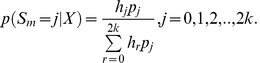
(9)

A point estimate of the sample allele frequency can then be obtained as *arg max_j_*{*p*(*S_m_* = *j* | *X*)}. As we often will be interested in SNP calling and genotype calling in all sites, and not only in sites in which the ancestral base is among the segregating nucleotides, inferences can be done using the folded, rather than the unfolded, frequency spectrum. To calculate the posterior probability, we then need to sum over foldings, and over assignments of derived and ancestral alleles:


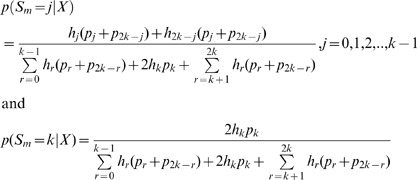
(10)

Finally, if we wish to take into account uncertainty in the assignments of ancestral and derived alleles, we need to sum over all possible pairs of segregating alleles:


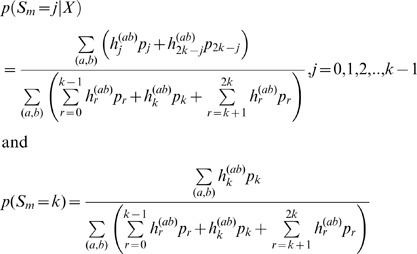
(11)

where 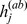
is the function *h_j_* calculated assuming *a* is derived and *b* is ancestral, and the sum is over all ordered pairs (a, b) ∈ *B* ^2^. There is here an implicit assumption of equal weighting of all possible alleles as ancestral and derived. The method could possibly be improved by using a more careful weighting using empirically derived proportions of segregating nucleotide pairs.

The expression given above can be used directly for SNP calling using a fixed cut-off for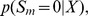 such as 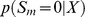
<0.05 or some lower value depending on how conservative one wants to be in calling SNPs.

If SNP calling has already been performed based on the same data, so that only sites expected to be variable are included in the analysis, estimation of allele frequencies should proceed by conditioning on the site being variable in the sample, by modifying the denominator in the expression above to reflex that zero probability is assigned to the event 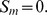
or 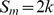. For example, Equation (11) becomes


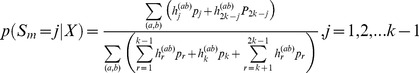
 and


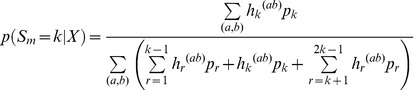
(12)

### Genotype probabilities

The framework derived above for allele frequency estimation and SNP calling can also be used for estimating individual genotype probabilities, leveraging information from all other individuals in the genotype call for a single individual. We will assume that the site has already been called to be variable with a SNP of a specific type with nucleotides *h* and *g*.

The posterior probability for a genotype for an individual, *d*, then becomes


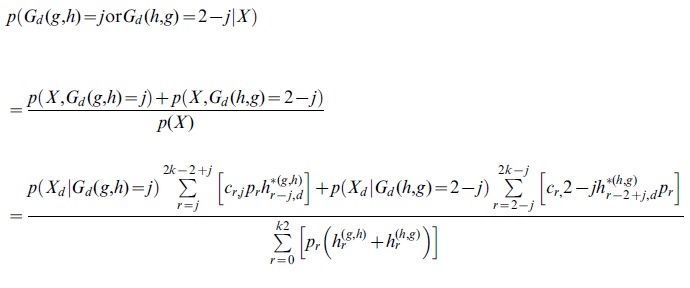
(13)

and


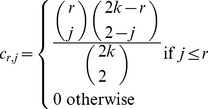


Here, the event 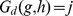
indicates that individual *d* has genotype *j* ∈ {0, 1, 2}, *j* indicating the number of derived allele, with *g* as the derived and *h* as the ancestral allele. 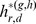
is the value of 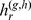 calculated for individuals (1, 2,..., *d*-1, *d*+1,.., *k*). This algorithm for estimation of genotype probabilities, therefore requires recalculation of the *h_j_* functions for all *k* possible subsets found by excluding one individual from the data.

We notice that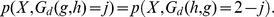 Furthermore, assuming symmetry in the probability of being ancestral and derived among nucleotides, 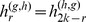
and 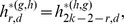 and, therefore the denominator can be calculated faster as


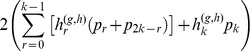
(14)

Likewise, the numerator becomes


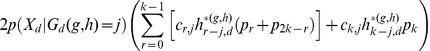
(15)

In cases where SNP calling precedes genotype calling, the summations in the numerator and denominator should be modified to appropriately condition on variability.

Again, specific weighting schemes for the pairs (*a, b*) could possibly be used to improve the estimates. Finally, we note that these expressions assume Hardy-Weinberg equilibrium.

### Incorporating external information regarding allele frequency

The algorithms described above have been developed assuming that no external information exists regarding allele frequencies. When that is not true, the algorithm can be modified to incorporate external estimates of the allele frequency.

Assume that we know the population allele frequency of the major allele, *f*, in the site. Then, assuming Hardy-Weinberg equilibrium, the marginal posterior for a particular genotype is


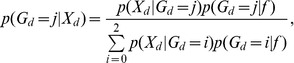
(16)

where, assuming Hardy-Weinberg Equilibrium.


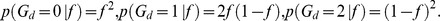
The allele frequency, *f*, will typically be based on estimates obtained from a larger set of sites. We can consider this another type of Empirical Bayes (EB) procedure in that a parameter of the prior for each individual is estimated jointly based on all individuals (and possibly other external data). We will use the maximum likelihood estimator of population allele frequency (not to be confused with sample allele frequency) described by [22] in any data applications in this paper. This approach may not work well when there are only very few individuals for which to estimate *f*. In such cases, it might work better to obtain joint ML estimates of genotypes from all individuals using an EM algorithm with *f* as the latent variable, to use a full Bayesian approach integrating the joint likelihood function all individuals over *f,* or to revert to the previously discussed methods which does not rely on estimation of *f*. When *k* is relatively large (e.g., >20), the EB procedure should provide marginal posteriors for the genotypes from each individual close to the ones that would have been obtained using a full Bayesian approach.

Similarly, we will use an estimator of *f* obtained for each site independently, but jointly for all individuals: the maximum likelihood estimator described by [8], [22]. For simulation purposes we will occasionally also use the estimator by [8], which is faster to calculate but may not be as accurate as the ML estimator. Determination of status as major or minor will be defined based on these estimates. Because of this we can also safely ignore the possibility that a site is invariable because the minor allele is fixed, and equate invariability to 0 < *S_m_* <2*k*.

We are then interested in obtaining


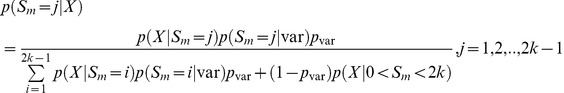
(17)

and *X* = (*X*_1_,…, *X_k_*) now is the vector of read data for all individuals. The variable ‘var’ indicates the event that the site is a variant, i.e. 0 < *S_m_* <2*k*. 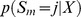 can then be estimated, using the same algorithm as described for calculation of the likelihood function, but with the following *Termination* step

*Termination*

Set *h_j,_* =  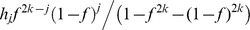 for *j* = 1,2,…,2*k*-1.

The posterior probabilities are then given by


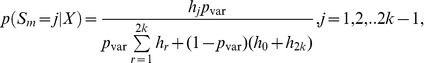
(18)

After completion of the algorithm, status of major and minor allele might then appropriately be re-assigned if this is used in the downstream inferences and if *p*(*S_m_* > *k* | *X*) >0.5. Alternatively, the results can be polarized with respect to ancestral and derived allele or be folded. The allele frequency can then be estimated as the value of *j* that maximizes 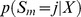, or inferences can, in most cases, more appropriately be made by summing over the posterior distribution of *S_m_*.

### Incorporating deviations from Hardy-Weinberg Equilibrium (HWE)

The EB estimator of allele frequency can also be modified to incorporate deviations from HWE. Assume that an inbreeding coefficient, *F_d_*, has been estimated for individual *d*, *d* = 1, 2, …, *k*. *F_d_* can take on both positive and negative values. Let 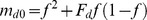, 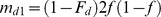, and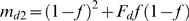. Then the following algorithm calculates the likelihood used in the EB estimation:

*Initialization:*

Set 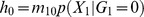,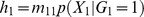,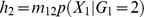, and *h_j_* = 0 for *j* = 3,4,…,2*k*.

*Recursion*

For *d* = 2, 3,…, *k:*

For *j* = 2*d*, 2*d*-1,…,2:

Set


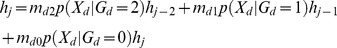


Set *h*_1_ *m_d_*_0_ *p*(*X_d_* | *G_d_* = 0)*h*_1_ + *m_d_*_1_ *p*(*X_d_* | *G_d_* = 1)*h*_0_

Set *h*_0_ = *m_d_*_0_ *p*(*X_d_* | *G_d_* = 0)*h*_0_

*Termination*

Set *h_j_* 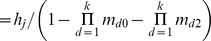 for *j* = 1,2,…,2*k*-1.

The posterior probabilities can then be evaluated as before.

### Simulations

To compare methods we conducted simulations under simplified assumptions. In all simulations, except if otherwise stated, we simulated data by allowing a Poisson distributed number of reads for each individual in each site independently of each other. The distribution of allele frequency (*x*) was assumed to be proportional to 1/*x* in the population. Each site is assumed to be variable with probability *p_var_*. Errors are introduced randomly an symmetrically among all bases. Genotype probabilities are calculated according to the model assuming known error rates. We also compared methods by examining their performance on real data. This was done using HapMap data with known genotypes (the reported genotype error rate is <0.1%).

## Results

In the Methods section, we described a likelihood function for **P**, i.e. we derived Pr(*X* | **P**), where *X* is all the sequencing data from multiple individuals and multiple sites. This is the likelihood function underlying the EM algorithm for estimating the SFS presented in [24]. [25] also developed a similar method, but could only analyze small sample sizes due to computational constraints. As shown in the Methods section, the likelihood function can be evaluated directly, using a dynamic programming algorithm, with computational time that is linear in the number of sites, and quadratic in the number of individuals. The function can be optimized using standard optimization algorithms, using analytical derivatives, to provide a maximum likelihood estimate of the SFS. This method provides a computational alternative to the method of [24] for obtaining maximum likelihood estimates of the SFS.

**Figure 1:**
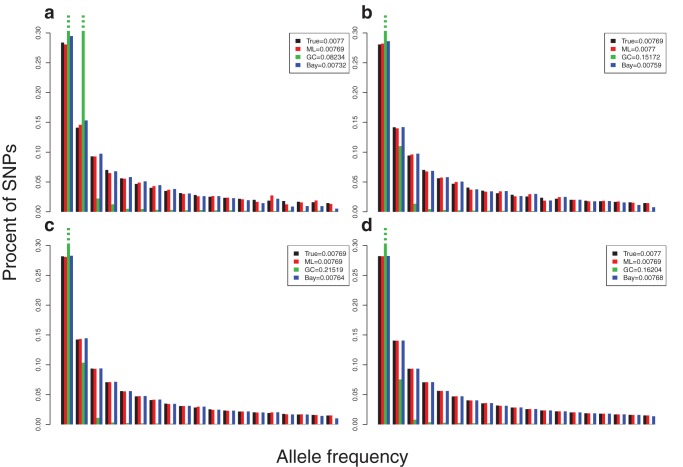
The distribution of true (True) and estimated unfolded SFS using the Maximum Likelihood method (ML) presented in the paper, genotype calling based on choosing the genotype with highest posterior probability (GC), and using the Bayesian procedure described in the text (Bay) in a sample from 50 MB 10 diploid individuals, where 2% of all SNPs are variable in the population and follow a distribution of allele frequencies, *p*, proportional to 1/*p*. An error rate of 0.5% is assumed. The mean sequencing depths are 1X (a), 3X (b), 5X (c), and 10X (d). The values presented in the figure legend box are the estimates of the proportion of sites that are variable in the sample.

To evaluate the method, we simulate data with known error rates (Fig. 1), mimicking the variation in sequencing depths observed in real data. The number of data points needed to provide good estimates depend both on the number of sites/SNPs analyzed, the number of individuals and on the sequencing depth. For example, 50 MB of data with 1% variable sites is sufficient to provide reasonable estimates even if the average sequencing depth is only 1X per individual (0.5X per chromosome). However, with only 10 MB, higher depth is needed and good estimates are first obtained with a depth of 3–5X.

To illustrate the difference between the new method and methods based directly on genotype calling, we compared with the case where the most likely genotype is chosen, with and without filtering of genotypes with low confidence (Figure 1). Clearly, simple genotype calling leads to an excess of singletons when no filtering is done. This problem can be partly corrected by using more conservative SNP calling procedures. But even in such cases, the SFS estimates tend to be poor from low frequency data. The effect is very similar to the one described [15], [16] in which they show that no simple cut-off method leads to unbiased estimates of the population genetic parameter *θ* ( = 4*N*µ where *N* is the population size and *μ* is the mutation rate) when using low or moderate coverage shotgun sequencing data. The same effect is observed for estimation of the SFS. Using filters in which only high confidence genotypes are called leads to new biases because it is easier to call homozygous than heterozygous individuals. This bias will affect different allele frequency categories differentially and lead to biases in the estimate of the SFS. [15], [16] argue that methods for estimating *θ* should instead take the inherent uncertainty in the data into account. The method developed here is a conceptual extension of this concept to the SFS.

### Inferences for individual sites

The method used for inferences of the SFS for a whole genome or for a large set of sites, can also be modified to make inferences for a single site [24]. The estimated SFS can be used as a prior for the allele frequency, and inferences regarding a particular site can then proceed using classical Bayesian procedures. The algorithmic details are provided in the Methods section. This method can be considered a Empirical Bayes method (e.g., [28]) as a large set of data points is being used to define a prior that subsequently can be applied to each data point. Figure 1 shows that, in average, the use of this procedure provides a distribution of allele frequencies that accurately reflects the true distribution of allele frequencies.

### SNP calling

**Figure 2:**
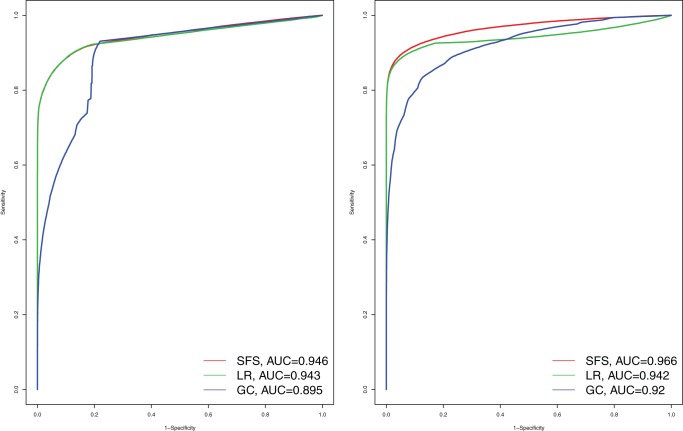
ROC curves for different SNP callers. Data for 10 individuals were simulated assuming a sequencing depth of 2 and a raw sequencing error rate of 1% (A) and (B) a depth of 5 and a raw sequencing error rate of 5%. The SFS method is the main method described in the text. The GC method is based on genotype calling using the genotype with the highest posterior probability. The LR method is based on a likelihood ratio test of the hypothesis that the allele frequency is zero. The SFS based method and the LR method have similar performance except for very high error rates, where the SFS tends to be somewhat better. Both methods in general perform much better than the GC method. The difference would even larger in larger panels of individuals. Simulations under other conditions can be found in Figure S1.

An algorithm similar to the one used for estimating the SFS can be used to make inferences for individual sites, and is described in the Methods section. The method proceeds by first estimating the SFS. The estimated distribution of allele frequencies, and the total frequency of SNPs in the sample (1 −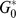), then provides priors in a Bayesian SNP caller similar to the one used in the 1000 Genomes project [7]. This is the approach outlined for SNP calling in [24]. We compare this type of SNP calling to two other methods: (1) traditional SNP calling based on observing at least *X* high quality reads of the minor allele, and (2) a likelihood ratio test based on testing the null hypothesis that the minor allele frequency is zero (Figure 2; Figure S1). The latter method is based on the likelihood function described in [11], [22], [23], [29].

We see that the Bayesian SNP caller is substantially better than traditional methods, but does only marginally better than the likelihood ratio tests (Figure 2; Figure S1). The use of prior information regarding allele frequencies only provide a marginal improvement. However, in the analyses of human data, or other data where large reference data sets are available, optimal SNP callers will include prior information from the reference data, possibly using methods related to imputation (e.g., [30], [31], [32], [33], [34]) as in the 1000 Genomes project [7]. For such methods, an important initial step is calculation of posterior probabilities for each SNP. Depending on the specifics of the implementation of the imputation methods, the methods described here for estimating sample allele frequencies may also be useful in the application of some imputation methods.

### Genotype calling

**Figure 3:**
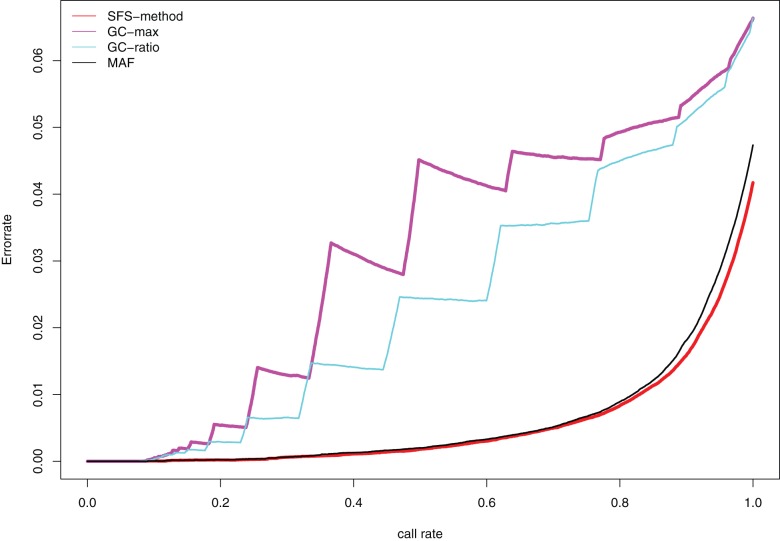
The error rate of different genotype callers for different call rates. The SFS-method is the method described in the main text. The MAF method is based on first obtaining a maximum likelihood estimate of the allele frequency, and then use the estimated allele frequency to define priors for genotype calling. The GC-max method is based on calling genotypes with highest posterior probability. The GC-ratio method is based on calling genotypes depending on the ratio of the likelihood for the most likely to second most likely genotype. The jagged behavior of some of the curves is a consequence of the discrete nature of the data, i.e. an individual contains a discrete number of copies of the minor allele. 10 individuals are simulated for 50,000 variable sites with a distribution of allele frequencies (*p*), proportional to 1/*p* with an error rate of 0.5%. Results for other error rates are shown in Figure S2.

The Methods section described a Bayesian method for genotype calling using the estimated SFS as a prior. In brief, it calculates the posterior probability of the genotype in each individual conditional on all data from both the focal individual and the other individuals in the sample. Information from other individuals can substantially improve genotype calling. This is illustrated using simulations in Figure 3 (see also Figure S2). Notice that the genotype calling accuracy is greatly improved compared to the case of just choosing the most likely genotype.

Again, in human data, and other data for which large reference data sets are available, these data should be incorporated for genotype calling. In fact, imputation based genotype calling will lead to a substantial increase in accuracy over other methods [7].

### Applications to data from 25 exomes

**Figure 4:**
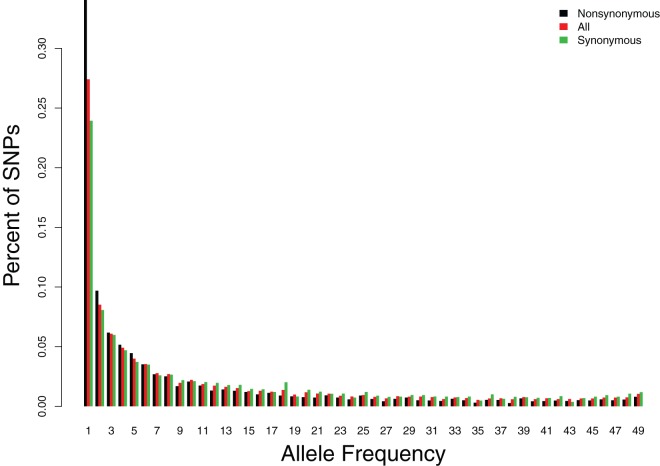
The unfolded site frequency spectrum from 25 Danish indivuduals. The data were previously analyzed in Yi *et al.* 2010.

To illustrate the use of these methods on real data, we analyzed previously published data from 25 Danish exomes [6]. The resulting frequency spectrum is depicted in Figure 4. As in [6] we find that nonsynonymous mutations show an excess of rare alleles compared to synonymous mutations, presumably due to slightly and weakly deleterious alleles.

The Methods section also describes a method for incorporating prior information regarding allele frequencies and for incorporating deviations from Hardy-Weinberg Equilibrium when estimation allele frequencies.

## Discussion

We have here developed a method for estimating the SFS that can be used for population genetic inferences. This method may also be used to define priors used in SNP calling and genotype calling leading to improved analyses of next-generation sequencing data.

The methods rely on accurate estimation of genotype likelihoods. Much research has been devoted to this (e.g. [10], [11]), and there is some hope that reasonably accurate genotype likelihoods eventually can be calculated for most sequencing platforms. However, it is worth emphasizing that inaccurate genotype likelihoods can lead to false inferences when applied in the present context. In real data, it can often be difficult to determine if genotype likelihoods have been calculated correctly. However, the improvements observed over simpler method when applied to real data, suggests that genotype likelihoods, as calculated by, for example, the SOAPsnp program ([11]) used here, provides sufficiently accurate genotype likelihoods to make the application of the new methods worthwhile.

Several of the methods presented here are similar to methods developed in parallel and recently published by [24] Li (2011). In particular, [24] provided an EM algorithm for estimating the SFS under the same model and [25] developed a method applicable to smaller samples. Our approach differs from these approaches by the use of a dynamic programming algorithm that makes the likelihood function accessible to direct fast evaluation and numerical optimization. Similar dynamic programming algorithm has previously been used in [8] and [26] for single site inferences. In addition, we show how to use the resulting estimated SFS for genotype calling. Our genotype caller differs from previous genotype callers by explicitly calculating the posterior probability of a genotype conditional on the data obtained from all individuals in the sample under a joint prior for the sample allele frequency. [26] presented closely related genotype callers based on inferences on single sites, also using a dynamic programming algorithm allowing calculation of joint allele frequencies. The SNP calling algorithm we use is identical to the one in [24]. We also present additional results on how to incorporate deviations from Hardy-Weinberg equilibrium when estimating allele frequencies, how to address issues regarding the folding of the frequency spectrum and how to incorporate external information regarding allele frequencies. In addition, we provide some simulation results evaluating the performance of the SNP callers, Genotype callers and SFS estimators.

A number of different methods have been proposed for estimating allele frequencies and the SFS from NGS data. In this paper we discuss the use of joint maximum likelihood estimates from multiple sites. This was also the approach taken by [24] and [25]. As illustrated in Figure 1, this approach will recover the true frequency spectrum when the modeling assumptions are correct. Methods based on estimating the allele frequency separately in each site will not generally have this property. [35] provided an alternative approach. The idea in this approach is to compare the inferred SFS based on genotype calling to the SFS obtained in other data that can be assumed not to have the types of biases introduced in NGS data. The extent of bias can then be quantified statistically, and used to correct SFS based on genotype calling in a larger data set. This approach may be preferable when the error structure is difficult to model, because it does not rely on such modeling. However, it requires the availability of accurate genotype calls from a large representative panel.

## Supporting Information

Figure S1 **ROC curves for different SNP callers.** Data for 10 individuals were simulated for different depths and error rates (*d* indicates depth and *e* is the reror rate). The SFS method is the main method described in the text. The GC method is based on genotype calling using the genotype with the highest posterior probability. The LR method is based on a likelihood ratio test of the hypothesis that the allele frequency is zero. larger panels of individuals.
(DOC)

Figure S2 **The error rate of different genotype callers for different call rates.** The SFS-method is the method described in the main text. The MAF method is based on first obtaining a maximum likelihood estimate of the allele frequency, and then use the estimated allele frequency to define priors for genotype calling. The GC-max method is based on calling genotypes with highest posterior probability. The GC-ratio method is based on calling genotypes depending on the ratio of the likelihood for the most likely to second most likely genotype. The jagged behavior of some of the curves is a consequence of the discrete nature of the data, i.e. an individual contains a discrete number of copies of the minor allele. 10 individuals are simulated for 50,000 variable sites with a distribution of allele frequencies (*p*), proportional to 1/*p* and with a varying error rate.
(DOC)
