# Supplementary figures and images for: SNP Calling, Genotype Calling, and Sample Allele Frequency Estimation from New-Generation Sequencing Data
Source: PLoS One. 2012 Jul 24;7(7):e37558. doi: 10.1371/journal.pone.0037558 (PMC3404070; doi:10.1371/journal.pone.0037558)

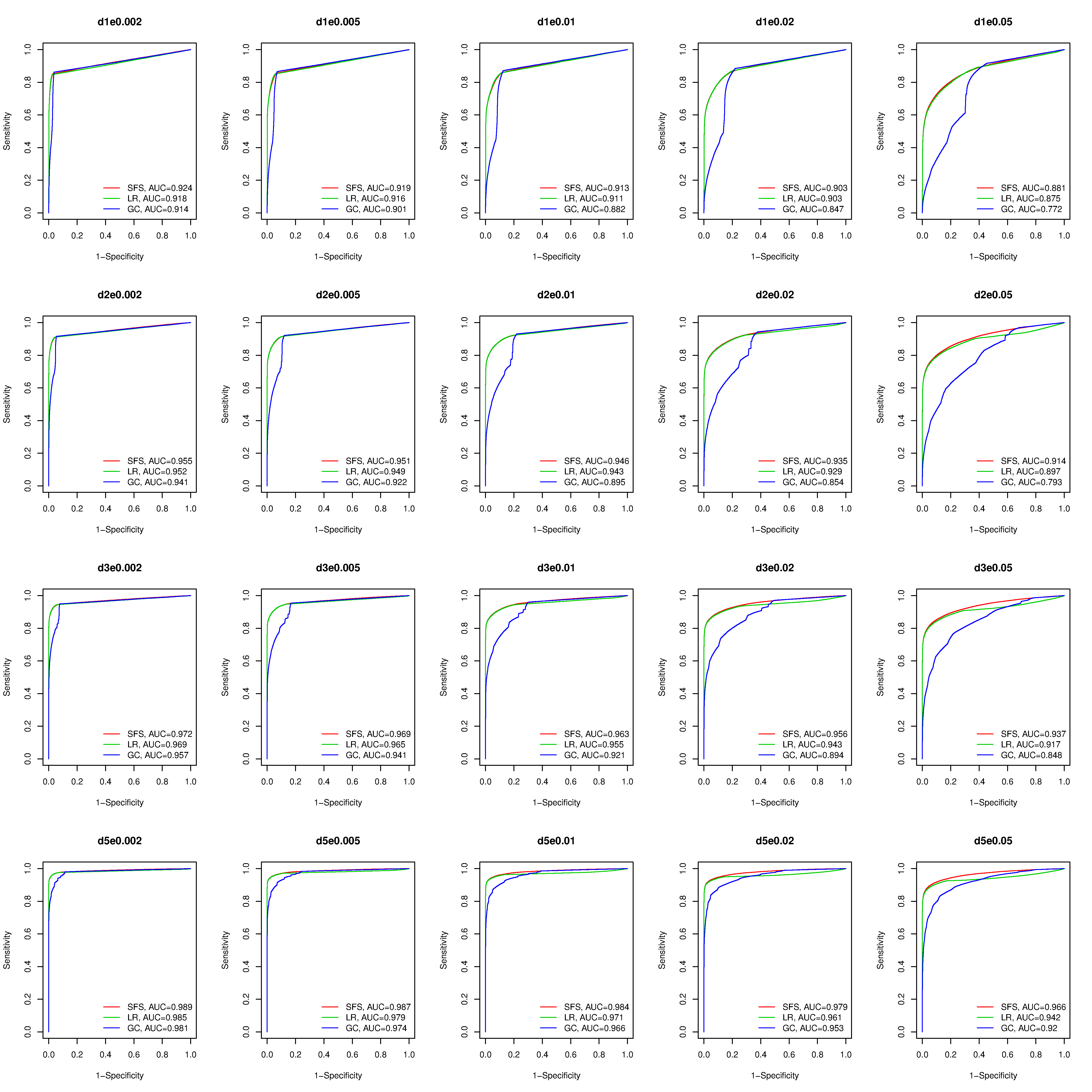

Supplement: Figure S1 — ROC curves for different SNP callers. Data for 10 individuals were simulated for different depths and error rates (d indicates depth and e is the reror rate). The SFS method is the main method described in the text. The GC method is based on genotype calling using the genotype with the highest posterior probability. The LR method is based on a likelihood ratio test of the hypothesis that the allele frequency is zero. larger panels of individuals. (DOC) [file pone.0037558.s001.doc]

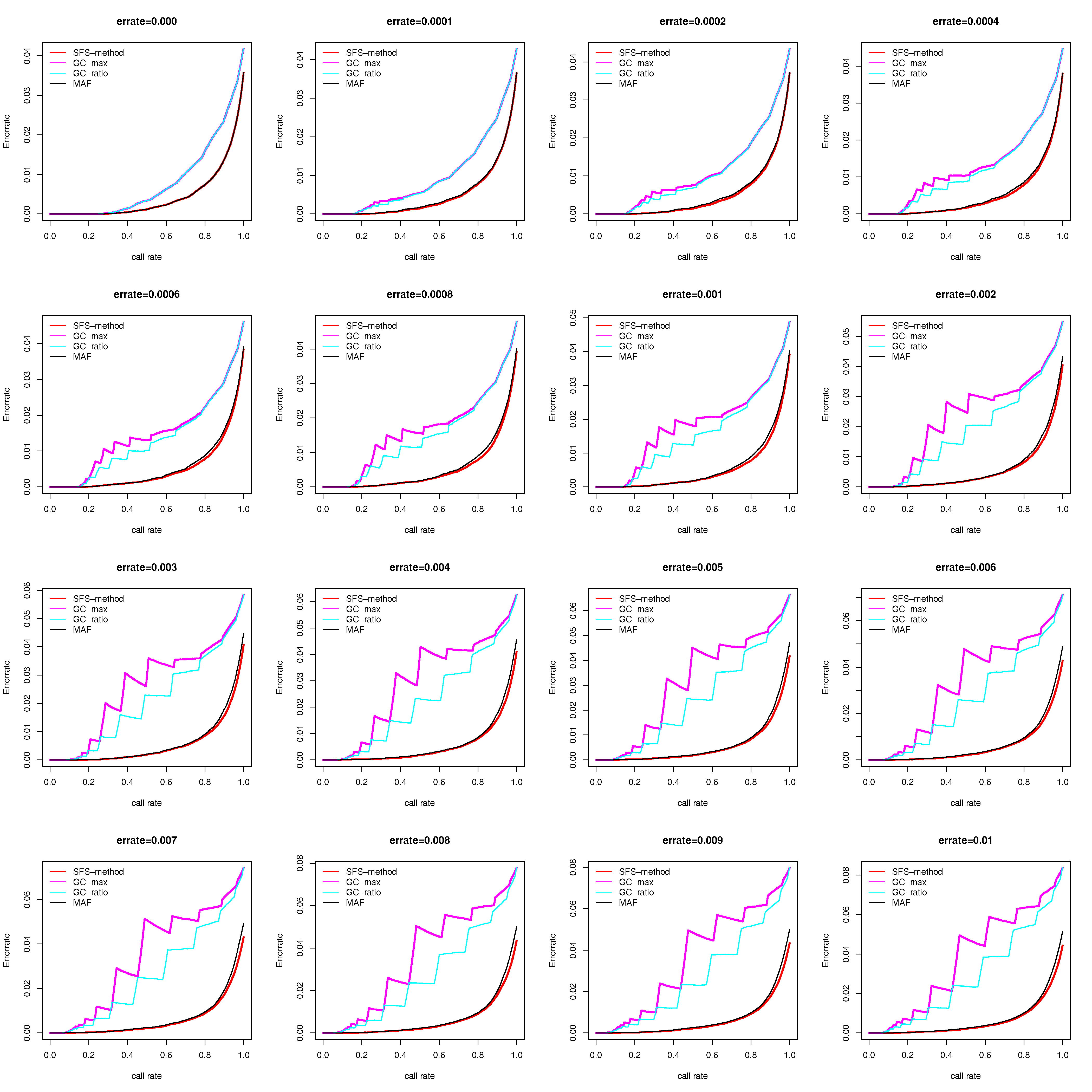

Supplement: Figure S2 — The error rate of different genotype callers for different call rates. The SFS-method is the method described in the main text. The MAF method is based on first obtaining a maximum likelihood estimate of the allele frequency, and then use the estimated allele frequency to define priors for genotype calling. The GC-max method is based on calling genotypes with highest posterior probability. The GC-ratio method is based on calling genotypes depending on the ratio of the likelihood for the most likely to second most likely genotype. The jagged behavior of some of the curves is a consequence of the discrete nature of the data, i.e. an individual contains a discrete number of copies of the minor allele. 10 individuals are simulated for 50,000 variable sites with a distribution of allele frequencies (p), proportional to 1/p and with a varying error rate. (DOC) [file pone.0037558.s002.doc]
